# Correlation Between Microbial Toxin Levels and Biochemical Markers of Organ Damage in ICU Patients

**DOI:** 10.7759/cureus.91870

**Published:** 2025-09-08

**Authors:** Harish Thummala, S Sangeeta, Bhagyashree K Bhuyar, Likhith Sai Kiran Rapeti, Ramesh Kandimalla

**Affiliations:** 1 Microbiology, Micro Labs, Guntur, IND; 2 Biochemistry, Government Medical College, Karimnagar, Karimnagar, IND; 3 Biochemistry, Koppal Institute of Medical Sciences, Koppal, IND; 4 Community Medicine, NRI Institute of Medical Sciences, Visakhapatnam, IND; 5 Biochemistry, Government Medical College, Narsampet, Narsampet, IND

**Keywords:** biochemical markers, endotoxin, exotoxin, icu, microbial toxins, organ injury, sepsis, sofa score

## Abstract

Background: Critically ill patients admitted to ICUs are at high risk of severe infections that may progress to sepsis and multi-organ dysfunction. Microbial toxins, such as endotoxins from Gram-negative bacteria and exotoxins from Gram-positive organisms, play a major role in the pathogenesis of systemic inflammation and tissue injury. While biochemical markers are routinely used to monitor organ function, their direct relationship with microbial toxin levels in ICU patients is not well established.

Objectives: To determine the correlation between circulating microbial toxin levels and biochemical indicators of organ damage in ICU patients with microbiologically confirmed infections.

Methods: A prospective observational study was conducted over 18 months in a tertiary care hospital ICU. A total of 120 adult patients (≥18 years) with confirmed bacterial or fungal infections were enrolled. Plasma endotoxin concentrations were measured using the limulus amebocyte lysate (LAL) chromogenic assay, with each sample analyzed in duplicate to ensure reproducibility. Intra- and inter-assay variability was maintained below 10% as per the manufacturer’s specifications, and polymyxin B inhibition was used in selected samples to confirm assay specificity. Gram-positive exotoxins were quantified using an enzyme-linked immunosorbent assay (ELISA), also performed in duplicate with appropriate positive and negative controls. Biochemical parameters included serum creatinine, alanine aminotransferase (ALT), aspartate aminotransferase (AST), lactate dehydrogenase (LDH), high-sensitivity troponin I, and total bilirubin. Organ dysfunction severity was evaluated using the Sequential Organ Failure Assessment (SOFA) scores. Data normality was assessed by the Shapiro-Wilk test, with Pearson’s correlation applied to normally distributed variables (creatinine, ALT, AST, hs-TnI) and Spearman’s correlation to skewed variables (bilirubin, LDH, endotoxin, exotoxin).

Results: The study cohort had a mean age of 54.8±13.2 years, with a male-to-female ratio of 1.4:1. Gram-negative organisms were identified in 68 patients (56.7%), Gram-positive in 38 (31.7%), and mixed infections in 14 (11.6%). Median endotoxin levels were significantly higher in Gram-negative infections compared to Gram-positive (Mann-Whitney U test, p<0.001). Endotoxin concentrations correlated strongly with serum creatinine (r=0.64, 95%CI: 0.48-0.75, p<0.001), total bilirubin (r=0.59, 95%CI: 0.42-0.72, p<0.001), and SOFA scores (r=0.71, 95%CI: 0.57-0.81, p<0.001). Exotoxin levels demonstrated significant correlations with LDH (r=0.55, 95%CI: 0.31-0.72) and high-sensitivity troponin I (r=0.48, 95%CI: 0.22-0.67, p=0.004). Patients with septic shock (n=42) exhibited markedly elevated toxin levels, deranged biochemical parameters, prolonged ICU stays (median: 12 days, IQR: 9-16), and mortality rates exceeding 50%. In multivariate logistic regression adjusting for SOFA scores and comorbidities, both endotoxin (OR=2.18) and exotoxin levels (OR=1.74) remained significant independent predictors of mortality and organ dysfunction severity.

Conclusion: Microbial toxin burden demonstrates a strong association with biochemical evidence of organ injury and disease severity in ICU patients. Combined toxin-biomarker profiling may have prognostic value for identifying high-risk patients, but its clinical application requires validation through larger multicentre studies with serial sampling before widespread adoption.

## Introduction

Severe infections in critically ill patients remain a major cause of morbidity and mortality worldwide, particularly among those admitted to intensive care units (ICUs) with sepsis and septic shock. The pathophysiology of sepsis involves a complex interplay between the host immune response and microbial factors, among which bacterial and fungal toxins play a pivotal role. These toxins act as potent triggers of systemic inflammation, contributing to endothelial dysfunction, microvascular injury, and ultimately multi-organ failure if not promptly addressed [1,2].

Endotoxins, primarily lipopolysaccharides (LPS) from the outer membrane of Gram-negative bacteria, are among the most studied microbial toxins. LPS binds to toll-like receptor 4 (TLR4) on immune cells, initiating a cascade of pro-inflammatory cytokine release, oxidative stress, and coagulation abnormalities [3,4]. In parallel, Gram-positive bacteria release exotoxins, such as toxic shock syndrome toxin-1 (TSST-1), alpha-toxin, and enterotoxins, which exert cytotoxic effects, disrupt cellular membranes, and act as superantigens, amplifying immune activation [5,6]. Fungal pathogens may also produce secondary metabolites and cell wall components, such as β-glucans, that modulate immune and inflammatory responses [7].

Biochemical markers, including serum creatinine, liver transaminases, lactate dehydrogenase (LDH), bilirubin, and cardiac troponins, are routinely employed to evaluate organ function in critically ill patients. These biomarkers not only provide information on the degree of organ injury but may also serve as prognostic indicators in sepsis and related conditions [8,9]. Previous studies have reported the correlations between elevated endotoxin activity and renal, hepatic, and cardiovascular dysfunction in septic patients [10,11]. However, the literature remains sparse regarding direct, quantitative correlations between specific microbial toxin levels and the biochemical markers of organ injury across different pathogen groups [12,13].

Understanding these associations is clinically important for early risk stratification in critically ill patients. Integrating toxin quantification with biochemical profiling may improve diagnostic precision, guide targeted therapeutic interventions, and support timely identification of patients at risk for rapid deterioration, particularly in high-burden settings where sepsis and mortality remain substantial.

The present study was designed to investigate the relationship between circulating microbial toxin levels and biochemical markers of organ damage in ICU patients with microbiologically confirmed infections. The primary objective was to assess the correlation between endotoxin concentrations and SOFA scores as an overall measure of disease severity. The secondary objectives were to evaluate the associations of toxin levels with organ-specific biochemical markers (serum creatinine, bilirubin, alanine transaminase (ALT), aspartate aminotransferase (AST), lactate dehydrogenase (LDH), high-sensitivity troponin-I (hs-TnI)), compare toxin profiles across pathogen groups, and examine their prognostic significance in relation to ICU length of stay and mortality.

## Materials and methods

Study design and setting

This prospective observational study was conducted in the ICU of Mahatma Gandhi Memorial (MGM) Hospital, Warangal, a tertiary care teaching hospital catering to a large patient population from both urban and rural areas. The study period spanned 18 months, from January 2023 to June 2024. The primary objective was to evaluate the relationship between circulating microbial toxin levels and biochemical markers of organ injury among critically ill patients with microbiologically confirmed infections. The study design followed established clinical research protocols for critical care biomarker evaluation. Ethical approval was obtained from the Institutional Ethics Committee of Kakatiya Medical College/MGM Hospital prior to initiation.

Study population

All adult patients aged 18 years and above admitted to the ICU during the study period with confirmed bacterial or fungal infections were screened for eligibility. Patients were included if they had positive microbiological culture reports from relevant clinical specimens, were admitted to the ICU within 24 hours of diagnosis, and provided informed consent either directly or via a legally authorised representative. Exclusion criteria comprised pre-existing chronic organ failure, such as advanced chronic liver disease, end-stage renal disease, or severe chronic heart failure; patients on long-term immunosuppressive therapy for conditions such as malignancy or autoimmune diseases; pregnant or lactating women; and those with incomplete clinical or laboratory data. This ensured that the observed biochemical changes could be attributed primarily to the acute infectious process and not confounded by pre-existing end-stage organ dysfunction.

Sample size determination

The sample size was calculated based on earlier reports indicating moderate correlation coefficients (r=0.4-0.6) between microbial toxin burden and biochemical evidence of organ dysfunction in septic ICU patients. Considering a power of 80% and a two-sided alpha error of 5%, the minimum sample size required was 108. To account for potential dropouts or incomplete data, a total of 120 patients were enrolled.

Clinical and demographic data collection

Demographic characteristics, comorbidities, primary infection site, causative organism, and antimicrobial susceptibility patterns were recorded in a structured proforma [12-15]. The severity of illness at admission was assessed using the Sequential Organ Failure Assessment (SOFA) score, which has been validated for predicting outcomes in sepsis and critical illness [14,15]. All clinical evaluations were performed by ICU physicians, and relevant laboratory investigations were initiated within the first 24 hours of ICU admission [10-14].

Microbiological analysis

Clinical specimens, including blood, urine, respiratory secretions, pus/wound swabs, and other body fluids, were collected under strict aseptic precautions. Microbiological identification was performed using standard culture techniques on appropriate media, followed by organism identification through automated systems such as VITEK® 2 Compact (bioMérieux, France). Antimicrobial susceptibility testing was carried out in accordance with Clinical and Laboratory Standards Institute (CLSI) guidelines [10-13].

Toxin quantification

Quantification of circulating microbial toxins was performed on plasma samples obtained within 24 hours of ICU admission. For Gram-negative bacterial infections, endotoxin levels were determined using the limulus amebocyte lysate (LAL) chromogenic assay (Lonza, Durham, NC), with results expressed in endotoxin units per millilitre (EU/mL). All assays were performed in duplicate runs, and intra- and inter-assay variability was maintained below 10%, in accordance with the manufacturer’s specifications. Each run included positive and negative controls, and selected samples were re-tested in the presence of polymyxin B to confirm assay specificity. For Gram-positive infections, exotoxins such as toxic shock syndrome toxin-1 (TSST-1) and staphylococcal enterotoxins were quantified using commercially available enzyme-linked immunosorbent assay (ELISA) kits, also performed in duplicate with appropriate controls. In cases of confirmed fungal infections, serum β-D-glucan levels were measured using the Fungitell® assay (Associates of Cape Cod, Inc., East Falmouth, MA), which has demonstrated utility as a non-culture-based diagnostic marker [10-13].

Biochemical marker assessment

Biochemical parameters reflecting organ injury were measured from venous blood samples collected concurrently with toxin assays. Renal function was assessed using serum creatinine (Jaffe’s kinetic method, Erba Mannheim XL-640 autoanalyzer). Hepatic injury markers included ALT and AST, estimated by the International Federation of Clinical Chemistry and Laboratory Medicine (IFCC) method without pyridoxal phosphate activation, and total bilirubin measured via the diazo reaction [10-13]. LDH activity, as an indicator of cellular injury, was determined by a kinetic UV assay. Cardiac injury was evaluated using hs-TnI, quantified by a chemiluminescent microparticle immunoassay (Abbott ARCHITECT i2000SR system, Lake County, LA). All biochemical analyses were completed within two hours of sample collection to prevent analyte degradation.

Statistical analysis

Statistical analyses were conducted using Statistical Product and Service Solutions (SPSS, version 26.0; IBM SPSS Statistics for Windows, Armonk, NY). Continuous variables were tested for normality using the Shapiro-Wilk test. Normally distributed variables (e.g., creatinine, ALT, AST, hs-TnI) were presented as mean ± SD, and non-normally distributed variables (e.g., bilirubin, LDH, endotoxin, exotoxin) as median with interquartile range (IQR). Pearson’s correlation coefficient was applied for normally distributed variables, while Spearman’s rank correlation was used for skewed variables. Group comparisons employed independent t-tests for normal data and Mann-Whitney U tests for non-normal data. To assess independent predictors of mortality and organ dysfunction severity, a multivariate logistic regression model was constructed, adjusting for age, sex, SOFA scores, and major comorbidities (diabetes, hypertension, chronic obstructive pulmonary disease (COPD)). Effect sizes were reported as adjusted odds ratios (ORs) with 95% confidence intervals (CIs). A two-tailed p-value <0.05 was considered statistically significant.

Ethical considerations

The study protocol was reviewed and approved by the Institutional Ethics Committee of Kakatiya Medical College/MGM Hospital. Written informed consent was obtained from each patient or their legal guardian prior to enrolment. Patient confidentiality was maintained throughout in accordance with the Declaration of Helsinki.

## Results

Patient demographics and clinical characteristics

A total of 120 patients were enrolled during the study period. The mean age of the study population was 54.8 ± 13.2 years, with 70 males (58.3%) and 50 females (41.7%). The most common comorbidities were type 2 diabetes mellitus in 51 patients (42.5%), systemic hypertension in 43 patients (35.8%), and COPD in 17 patients (14.2%). The predominant primary infection sites were the respiratory tract in 56 patients (46.7%), bloodstream in 36 patients (30.0%), urinary tract in 19 patients (15.8%), and surgical/wound infections in nine patients (7.5%) (Table 1).

**Table 1:** Baseline demographic and clinical characteristics (n=120) Independent t-test for continuous variables and chi-square test for categorical variables. COPD: chronic obstructive pulmonary disease; SOFA: Sequential Organ Failure Assessment

Parameter	Total (n=120)	Male (n=70)	Female (n=50)	p-value	t-value
Age (years, mean ± SD)	54.8 ± 13.2	55.9 ± 12.8	53.3 ± 13.7	0.38	0.88
Diabetes mellitus (%)	51 (42.5%)	32 (45.7%)	19 (38.0%)	0.41	–
Hypertension (%)	43 (35.8%)	24 (34.3%)	19 (38.0%)	0.69	–
COPD (%)	17 (14.2%)	11 (15.7%)	6 (12.0%)	0.58	–
SOFA score (median, IQR)	8 (6–12)	8 (6–12)	7 (6–11)	0.42	–
ICU stay (days, median, IQR)	9 (6–14)	9 (6–15)	8 (5–13)	0.47	–

Microbiological profile and toxin levels

Gram-negative organisms were isolated in 68 patients (56.7%), Gram-positive in 38 (31.7%), and mixed bacterial infections in 14 (11.6%). Among Gram-negative pathogens, *Klebsiella pneumoniae* (22.5%), *Escherichia coli* (20.0%), and *Acinetobacter baumannii* (14.2%) were most frequently identified. Among Gram-positive isolates, *Staphylococcus aureus* (including methicillin-resistant *S. aureus *(MRSA)) was predominant (21.7%), followed by *Enterococcus faecalis* (10.0%).

Median plasma endotoxin levels were significantly higher in Gram-negative infections (0.92 EU/mL; IQR: 0.70-1.25) compared with Gram-positive infections (0.18 EU/mL; IQR: 0.10-0.26; Mann-Whitney U test, p<0.001). Conversely, exotoxin levels were elevated in Gram-positive infections (3.42 ng/m; IQR: 2.85-4.12) compared with Gram-negative cases (0.21 ng/mL; IQR: 0.14-0.32; p<0.001). Patients with mixed infections demonstrated intermediate values for both endotoxin (0.74 EU/mL; IQR: 0.62-0.92) and exotoxin (2.12 ng/mL; IQR: 1.45-2.88), with statistically significant differences versus single-pathogen groups (p<0.05) (Table 2).

**Table 2:** Microbial toxin levels by the pathogen group Independent t-test or Mann–Whitney U test where appropriate.

Pathogen Group	Endotoxin (EU/mL, median (IQR))	p-value	t-value	Exotoxin (ng/mL, median (IQR))	p-value	t-value
Gram-negative (n=68) (56.7%)	0.92 (0.70–1.25)	<0.001	14.72	0.21 (0.14–0.32)	<0.001	12.94
Gram-positive (n=38) (31.7%)	0.18 (0.10–0.26)	–	–	3.42 (2.85–4.12)	–	–
Mixed (n=14) (11.7%)	0.74 (0.62–0.92)	0.012	2.57	2.12 (1.45–2.88)	0.019	2.40

**Figure 1:**
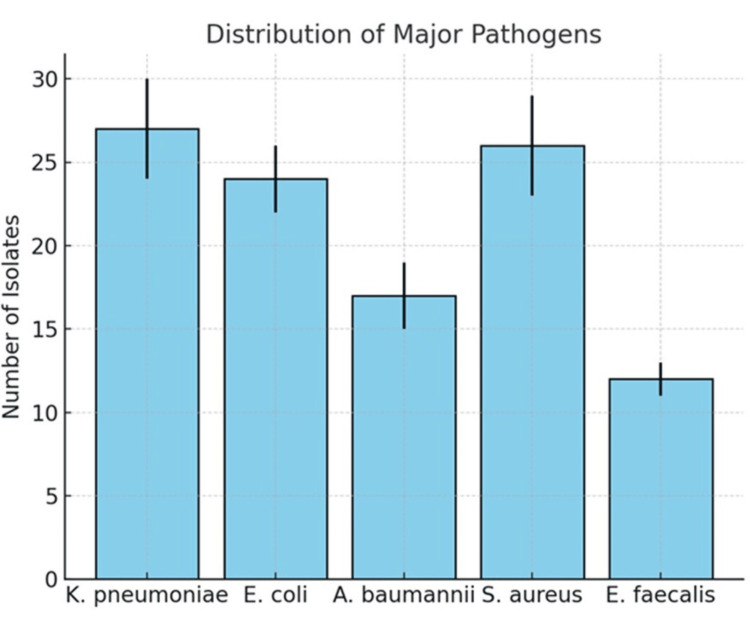
Distribution of major pathogens (n=120) The frequency of the five most common organisms identified in the study population. *Klebsiella pneumoniae *and *Staphylococcus aureus* were the leading isolates, followed by *Escherichia coli*, *Acinetobacter baumannii*, and *Enterococcus faecalis*. Error bars represent the standard error of the mean (SEM) for each group.

The distribution of major pathogens isolated from ICU patients (n=120) is shown in Figure 1. Among Gram-negative organisms, *K. pneumoniae* was the most frequently encountered isolate, accounting for 27 cases (22.5%), followed closely by *E. coli *with 24 cases (20.0%). *A. baumannii* was identified in 17 patients (14.2%), reflecting its recognized role as an opportunistic and often multidrug-resistant pathogen in the critical care setting.

Among Gram-positive organisms, *S. aureus *(including MRSA) was predominant, being detected in 26 patients (21.7%). This highlights the continuing clinical burden of staphylococcal infections in critically ill populations. *E. faecalis* accounted for 12 isolates (10.0%), which, while less frequent than other pathogens, remains clinically significant given its association with multidrug resistance and healthcare-associated infections.

Overall, Gram-negative bacteria collectively represented the majority of isolates (56.7%), underscoring their dominant contribution to severe infections in the ICU setting. These findings are consistent with regional and global reports that emphasize the predominance of Gram-negative sepsis while acknowledging the continued impact of Gram-positive organisms, such as *S. aureus* and *E. faecalis* in critically ill patients.

Correlation between toxin levels and biochemical markers

A detailed correlation analysis was performed to explore the relationship between circulating microbial toxin levels and biochemical evidence of organ injury in ICU patients. Endotoxin concentrations demonstrated a robust positive correlation with markers of renal and hepatic dysfunction. Specifically, higher endotoxin levels were significantly associated with increased serum creatinine (r=0.64, 95% CI: 0.48-0.75; p<0.001), indicating impaired renal function, and with elevated total bilirubin (r=0.59, 95% CI: 0.42-0.72; p<0.001), reflecting hepatocellular or cholestatic injury. Furthermore, endotoxin levels showed the strongest correlation with global disease severity, as measured by the SOFA score (r=0.71, 95% CI: 0.57-0.81; p<0.001), suggesting that endotoxemia is a major contributor to multi-organ dysfunction in critically ill patients.

In contrast, exotoxin concentrations were more closely linked to cytolytic and cardiotoxic injury. Significant correlations were observed with LDH (r=0.55, 95% CI: 0.31-0.72; p=0.002), a marker of widespread cellular injury, and hs-TnI (r=0.48, 95% CI: 0.22-0.67; p=0.004), reflecting myocardial damage. These findings highlight that Gram-positive exotoxins exert distinct pathological effects compared to Gram-negative endotoxins, with a tendency to induce more pronounced cytotoxic and cardiotoxic responses.

The differential correlation patterns underscore the pathogen-specific injury signatures observed in this study. While endotoxin-driven injury was most evident in renal and hepatic dysfunction, exotoxin-driven effects were more pronounced in markers of cellular and cardiac damage. Together, these results strengthen the argument for combined toxin-biomarker profiling, which not only captures the burden of infection but also provides insight into the specific organ systems most at risk (Table 3).

**Table 3:** Correlation of microbial toxin levels with biochemical markers Pearson’s correlation coefficient for normally distributed variables; Spearman’s rank correlation for skewed variables. ALT: alanine transaminase; AST: aspartate aminotransferase; LDH: lactate dehydrogenase; hs-TnI: high-sensitivity troponin-I; SOFA: Sequential Organ Failure Assessment

Biochemical Marker	Endotoxin (r)	p-value	Exotoxin (r)	p-value
Serum creatinine	0.64	<0.001	0.28	0.045
Total bilirubin	0.59	<0.001	0.21	0.098
ALT	0.38	0.012	0.29	0.041
AST	0.41	0.008	0.31	0.036
LDH	0.52	0.003	0.55	0.002
hs-TnI	0.37	0.015	0.48	0.004
SOFA score	0.71	<0.001	0.62	<0.001

Correlation between toxin levels and biochemical evidence of organ injury

The correlation analysis revealed clear and clinically meaningful associations between microbial toxin concentrations and biochemical indicators of organ dysfunction among critically ill patients. Endotoxin burden was strongly linked to both renal and hepatic impairment. Elevated endotoxin levels were positively correlated with serum creatinine (r=0.64; p<0.001), reflecting compromised kidney function, and with total bilirubin (r=0.59; p<0.001), indicating disturbances in hepatic clearance and hepatocellular injury. In addition, endotoxin concentrations demonstrated the strongest relationship with overall disease severity, as captured by SOFA scores (r=0.71; p<0.001). This suggests that endotoxemia not only contributes to individual organ dysfunction but also acts as a significant driver of multi-organ failure in the ICU setting.

Exotoxin concentrations, by contrast, showed a different injury pattern, being more closely associated with markers of cellular and cardiac damage. Strong positive correlations were observed with LDH (r=0.55; p=0.002), an enzyme released during widespread cell injury, and with hs-TnI (r=0.48; p=0.004), a biomarker of myocardial injury. These associations point to the cytotoxic and cardiotoxic nature of Gram-positive exotoxins, which appear to exert their effects through direct tissue injury and myocardial stress.

**Figure 2:**
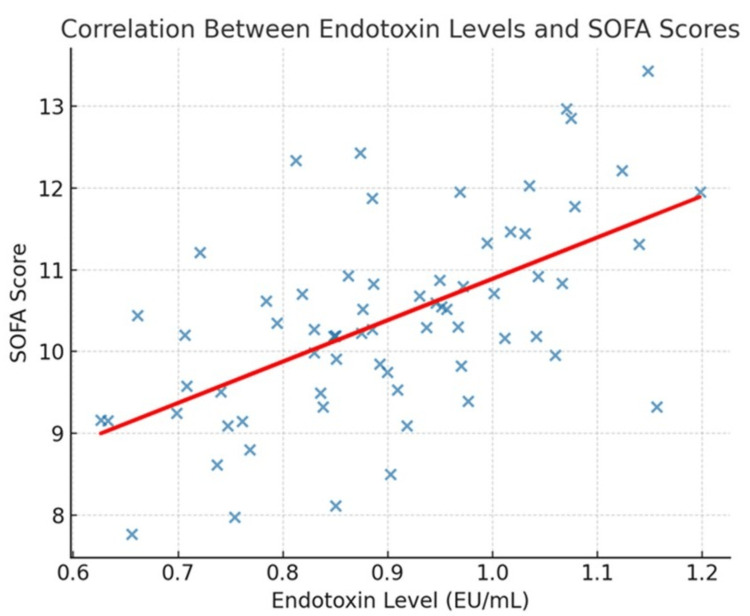
Scatter plot showing the correlation between endotoxin levels and SOFA scores (n=120) The scatter plot depicts the relationship between endotoxin levels (expressed in EU/mL) and organ dysfunction severity assessed by SOFA score. Each data point represents an individual patient, and the fitted regression line demonstrates a strong positive correlation (r=0.71; p<0.001), indicating that higher endotoxin burden is associated with greater organ dysfunction. SOFA: Sequential Organ Failure Assessment

Together, these findings highlight that endotoxins and exotoxins exhibit distinct biochemical signatures of organ involvement. While endotoxins primarily drive renal and hepatic dysfunction, exotoxins are more closely tied to cytolytic and cardiac damage. This pathogen-specific pattern supports the integration of toxin quantification with routine biomarker testing as a potential prognostic approach. Such combined profiling may help identify patients at heightened risk of rapid deterioration, offering opportunities for timely intervention in critical care (Figure 2).

To facilitate clinical interpretation, the differential patterns of organ involvement associated with Gram-negative and Gram-positive infections are summarized in Table 4. This overview highlights how endotoxins were predominantly linked to renal and hepatic dysfunction, whereas exotoxins were more closely associated with cytotoxic and myocardial injury, with mixed infections showing intermediate patterns. Such a summary may aid in translating laboratory findings into bedside monitoring strategies.

**Table 4:** Summary of pathogen-specific toxin–biomarker associations and clinical implications LPS: lipopolysaccharide; LDH: lactate dehydrogenase; hs-TnI: high-sensitivity troponin-I; SOFA: Sequential Organ Failure Assessment; TSST-1: toxic shock syndrome toxin-1

Pathogen group	Predominant toxins	Strongest biomarker correlations	Clinical implications
Gram-negative	Endotoxins (LPS)	Serum creatinine, Total bilirubin, SOFA	Renal and hepatic dysfunction; global severity burden
Gram-positive	Exotoxins (TSST-1, enterotoxins, cytotoxins)	LDH, hs-TnI	Cytolysis and myocardial injury
Mixed infections	Endotoxins + Exotoxins	Intermediate elevations across markers	Combined multi-organ involvement

Outcomes and prognostic implications

Patients who developed septic shock (n=42) exhibited substantially higher circulating microbial toxin concentrations compared to non-shock patients. Median endotoxin levels in this group were 1.24 EU/mL, while exotoxin concentrations reached 4.02 ng/mL. These elevations were paralleled by significantly higher biochemical markers of renal, hepatic, and myocardial injury, reflecting widespread multi-organ dysfunction. Clinically, patients with shock required prolonged ICU support, with a median stay of 12 days (IQR: 9-16), compared to shorter durations among those without shock.

**Figure 3:**
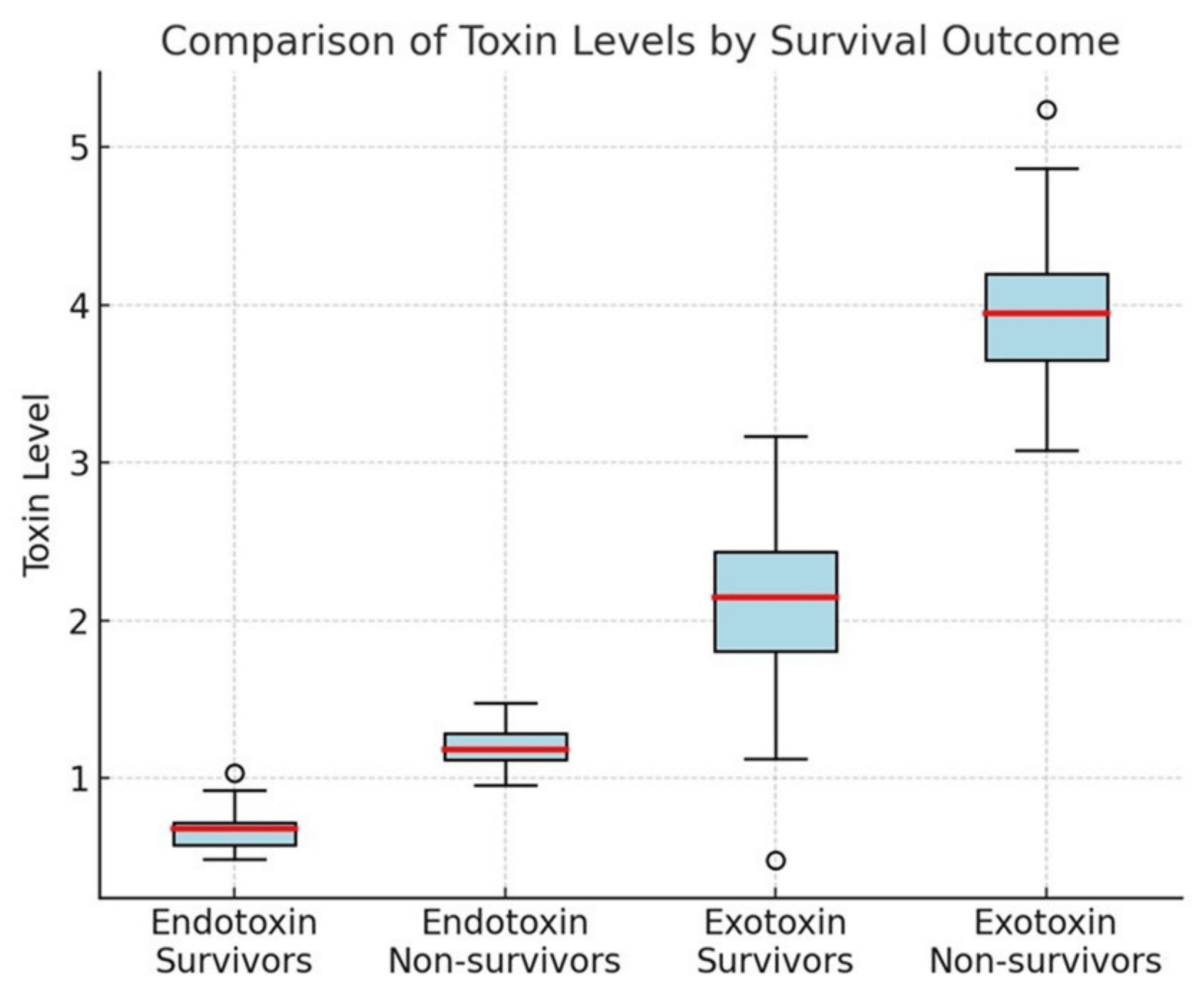
Box-and-whisker plots comparing endotoxin and exotoxin levels between survivors (n=78) and non-survivors (n=42)

Mortality outcomes demonstrated a striking difference between groups: over half of patients with septic shock (54.8%) died during ICU admission, compared to 21.3% of those without shock (p<0.001; χ²=14.56). When survivors and non-survivors were compared directly, non-survivors consistently showed elevated toxin concentrations, indicating that a higher microbial toxin burden was associated with poorer clinical outcomes. These differences in toxin distributions are depicted in Figure 3, which illustrates the clear separation of endotoxin and exotoxin levels between survivors and non-survivors.

To determine whether microbial toxins were independent predictors of outcome, a multivariate logistic regression model was performed, adjusting for SOFA scores and major comorbidities (diabetes mellitus, hypertension, COPD). After adjustment, both endotoxin levels (adjusted OR=2.18, 95% CI: 1.32-3.62; p=0.002) and exotoxin levels (adjusted OR=1.74, 95% CI: 1.11-2.89; p=0.018) remained significant predictors of mortality. These results confirm that toxin concentrations provide prognostic information beyond traditional severity indices and comorbid conditions.

Overall, the findings suggest that microbial toxin burden is not merely a marker of infection severity but may play a causal and independent role in driving organ failure, prolonged ICU stay, and increased risk of death. Incorporating toxin assays into critical care evaluation could therefore aid in early identification of high-risk patients and guide timely therapeutic interventions.

## Discussion

This prospective observational study demonstrated a significant association between circulating microbial toxin levels and biochemical markers of organ injury in critically ill patients with confirmed bacterial or fungal infections. Both Gram-negative endotoxins and Gram-positive exotoxins showed distinct patterns of correlation with biochemical parameters, suggesting that toxin quantification may provide clinically valuable prognostic information when combined with standard laboratory markers.

The predominance of Gram-negative organisms in our cohort, particularly *K. pneumoniae *and *E. coli*, aligns with earlier ICU-based studies from India and other low- and middle-income countries, where Gram-negative sepsis remains a major challenge due to high antimicrobial resistance rates [16,17]. Elevated endotoxin levels in these patients were strongly correlated with serum creatinine and bilirubin, indicating a direct link between Gram-negative infection burden and renal and hepatic dysfunction. This finding is consistent with prior experimental and clinical evidence that endotoxemia triggers a systemic inflammatory cascade, leading to microcirculatory alterations, tubular injury, and cholestasis [4,18]. The distribution pattern of these pathogens in our ICU cohort is depicted in Figure 3.

In contrast, Gram-positive exotoxins, particularly staphylococcal superantigens and cytotoxins, demonstrated stronger correlations with LDH and hs-TnI. This suggests that Gram-positive sepsis may exert more pronounced cytotoxic and cardiotoxic effects, possibly via superantigen-mediated immune activation and toxin-induced membrane damage [5,6]. Similar observations have been reported in animal models and select human studies, although the magnitude of correlation in our data indicates a potential clinical application for early cardiac risk stratification in Gram-positive sepsis [19].

The relationship observed between toxin levels and SOFA scores in our study indicates that higher microbial toxin concentrations were associated with greater overall organ dysfunction. However, since both toxin levels and organ dysfunction were measured at a single time point, these findings reflect an association rather than causation, and temporal relationships cannot be inferred. The strong positive correlation between endotoxin levels and SOFA scores (r=0.71; p<0.001) reinforces the hypothesis that toxin burden may serve as an independent predictor of disease severity. Prior research has also suggested that endotoxin activity assays can predict adverse outcomes and guide targeted interventions, such as polymyxin B hemoperfusion in selected septic patients [20,21]. This finding is further supported by the scatter plot shown in Figure 1.

Patients with septic shock in our cohort exhibited markedly elevated toxin concentrations, higher biochemical marker derangements, and substantially increased mortality rates. This finding underscores the potential role of early toxin measurement in identifying high-risk patients. Integrating toxin assays into ICU diagnostic protocols could allow earlier initiation of supportive measures and, where appropriate, adjunctive therapies aimed at toxin neutralization [10,11]. Comparative toxin distributions between survivors and non-survivors are shown in Figure 2. The comparison of endotoxin and exotoxin levels between survivors and non-survivors is depicted in Figure 2. These differences in toxin burden and outcomes are clearly demonstrated in Figure 2.

The study also highlights important differences between pathogen groups in terms of their biochemical injury signatures. While renal and hepatic impairment predominated in Gram-negative infections, cytolysis and myocardial injury markers were more pronounced in Gram-positive cases. This pathogen-specific injury pattern may have therapeutic implications, including targeted monitoring of vulnerable organ systems depending on the identified pathogen and toxin profile.

Our findings must be interpreted in light of certain limitations. The study was conducted in a single tertiary care centre, which may limit generalisability. Additionally, toxin quantification was performed at a single time point; serial measurements might have provided deeper insight into dynamic changes and their temporal relationship with organ dysfunction. The relatively small subgroup of fungal infections also limited pathogen-specific statistical comparisons for β-D-glucan. Despite these limitations, the strength of associations and statistical significance of our findings suggest clinical utility.

Limitations

This study has certain limitations that should be considered when interpreting the findings. First, it was conducted in a single tertiary care center, which may limit the generalizability of results to other healthcare settings with differing patient populations, pathogen prevalence, and treatment protocols. Second, toxin measurements were performed at a single time point within the first 24 hours of ICU admission. Serial monitoring would have provided a clearer picture of the dynamic relationship between toxin burden, biochemical alterations, and clinical outcomes. Third, although patients with advanced pre-existing organ failure were excluded, undetected subclinical dysfunction could still have influenced biochemical marker levels. Fourth, the relatively small number of fungal infections reduced the power to perform detailed pathogen-specific analyses for β-D-glucan and related markers. Fifth, the inclusion criteria were restricted to culture-positive patients, which may have introduced selection bias by excluding individuals with culture-negative sepsis who might also exhibit elevated toxin levels. Sixth, assay-related factors, such as potential cross-reactivity (e.g., interference of β-glucans in LAL-based endotoxin testing), could have affected the accuracy of toxin measurements. Finally, resource constraints limited the use of advanced molecular methods such as mass spectrometry-based toxin profiling, which may offer greater specificity and sensitivity compared to immunoassay-based approaches.

Future multicentre studies with larger and more diverse patient cohorts, serial toxin measurements, and incorporation of advanced detection platforms are warranted to validate and strengthen these findings.

## Conclusions

This study demonstrates an association between circulating microbial toxin levels and biochemical markers of organ dysfunction in critically ill patients. Endotoxin concentrations were most closely linked with renal and hepatic impairment, whereas exotoxin levels showed stronger associations with cellular and myocardial injury. Both toxin groups were also associated with higher SOFA scores, suggesting a potential role as indicators of disease severity.

The integration of microbial toxin assays with standard biochemical monitoring may have prognostic value for early risk assessment in the ICU. Recognition of pathogen-specific toxin-biomarker patterns could help identify organ systems at greatest risk, thereby informing monitoring priorities and treatment strategies. However, these findings are based on single-center, cross-sectional data. Larger multicentre studies with serial toxin measurements and external validation are needed before clinical adoption.

## References

[1] Singer M, Deutschman CS, Seymour CW (2016). The Third International Consensus Definitions for Sepsis and Septic Shock (Sepsis-3). JAMA.

[2] Hotchkiss RS, Moldawer LL, Opal SM, Reinhart K, Turnbull IR, Vincent JL (2016). Sepsis and septic shock. Nat Rev Dis Primers.

[3] Beutler B, Rietschel ET (2003). Innate immune sensing and its roots: the story of endotoxin. Nat Rev Immunol.

[4] Opal SM (2010). 14-24: Endotoxins and other sepsis triggers. Endotoxemia and Endotoxin Shock: Disease, Diagnosis and Therapy.

[5] Krakauer T (2019). Staphylococcal superantigens: pyrogenic toxins induce toxic shock. Toxins (Basel).

[6] Spaan AN, van Strijp JA, Torres VJ (2017). Leukocidins: staphylococcal bi-component pore-forming toxins find their receptors. Nat Rev Microbiol.

[7] Brown GD, Denning DW, Gow NA, Levitz SM, Netea MG, White TC (2012). Hidden killers: human fungal infections. Sci Transl Med.

[8] Pierrakos C, Vincent JL (2010). Sepsis biomarkers: a review. Crit Care.

[9] Prowle JR, Molan MP, Hornsey E, Bellomo R (2012). Measurement of renal blood flow by phase-contrast magnetic resonance imaging during septic acute kidney injury: a pilot investigation. Crit Care Med.

[10] Marshall JC, Foster D, Vincent JL (2004). Diagnostic and prognostic implications of endotoxemia in critical illness: results of the MEDIC study. J Infect Dis.

[11] Saito H, Sherwood ER, Varma TK, Evers BM (2003). Effects of aging on mortality, hypothermia, and cytokine induction in mice with endotoxemia or sepsis. Mech Ageing Dev.

[12] Vincent JL, Opal SM, Marshall JC, Tracey KJ (2013). Sepsis definitions: time for change. Lancet.

[13] Pierrakos C, Velissaris D, Bisdorff M, Marshall JC, Vincent JL (2020). Biomarkers of sepsis: time for a reappraisal. Crit Care.

[14] Jones AE, Trzeciak S, Kline JA (2009). The sequential organ failure assessment score for predicting outcome in patients with severe sepsis and evidence of hypoperfusion at the time of emergency department presentation. Crit Care Med.

[15] Moreno R, Rhodes A, Piquilloud L (2023). The sequential organ failure assessment (SOFA) score: has the time come for an update?. Crit Care.

[16] Walia K, Sharma M, Vijay S, Shome BR (2019). Understanding policy dilemmas around antibiotic use in food animals & offering potential solutions. Indian J Med Res.

[17] Savanur SS, Gururaj H (2019). Study of antibiotic sensitivity and resistance pattern of bacterial isolates in intensive care unit setup of a tertiary care hospital. Indian J Crit Care Med.

[18] Laupland KB, Kirkpatrick AW, Church DL, Ross T, Gregson DB (2004). Intensive-care-unit-acquired bloodstream infections in a regional critically ill population. J Hosp Infect.

[19] Bayer AS, Schneider T, Sahl HG (2013). Mechanisms of daptomycin resistance in Staphylococcus aureus: role of the cell membrane and cell wall. Ann N Y Acad Sci.

[20] Iba T, Gando S, Thachil J (2014). Anticoagulant therapy for sepsis-associated disseminated intravascular coagulation: the view from Japan. J Thromb Haemost.

[21] Cruz DN, Antonelli M, Fumagalli R (2009). Early use of polymyxin B hemoperfusion in abdominal septic shock: the EUPHAS randomized controlled trial. JAMA.

